# “Systems seem to get in the way”: a qualitative study exploring experiences of accessing and receiving support among informal caregivers of people living with chronic kidney disease

**DOI:** 10.1186/s12882-023-03444-3

**Published:** 2024-01-03

**Authors:** Chelsea Coumoundouros, Paul Farrand, Robbert Sanderman, Louise von Essen, Joanne Woodford

**Affiliations:** 1 Healthcare Sciences and e-Health, Department of Women’s and Children’s Health, Uppsala University, Dag Hammarskjölds väg 14B, Uppsala, 751 05 Sweden; 2 Clinical Education, Development and Research (CEDAR); Psychology, University of Exeter, Exeter, UK; 3 Department of Health Psychology, University Medical Center Groningen, University of Groningen, Groningen, Netherlands

**Keywords:** Informal caregiver, Chronic kidney disease, Support, Unmet needs, Social networks, Thematic analysis

## Abstract

**Background:**

The well-being of informal caregivers of people living with chronic kidney disease is influenced by their experiences with support, however, few studies have focused on exploring these experiences. This study aimed to explore informal caregivers’ experiences accessing and receiving support while caring for someone living with chronic kidney disease.

**Methods:**

Informal caregivers of people living with chronic kidney disease (n = 13) in the United Kingdom were primarily recruited via community organisations and social media adverts to participate in semi-structured interviews. Interviews explored support needs, experiences of receiving support from different groups (e.g. healthcare professionals, family/friends), and barriers and facilitators to accessing support. Support was understood as including emotional, practical, and informational support. Data were analysed using reflexive thematic analysis.

**Results:**

Three themes were generated: (1) “Systems seem to get in the way” – challenges within support systems, illustrating the challenges informal caregivers encountered when navigating complex support systems; (2) Relying on yourself, describing how informal caregivers leveraged their existing skills and networks to access support independently, while recognising the limitations of having to rely on yourself to find support; and (3) Support systems can “take the pressure off”, showing how support systems were able to help informal caregivers cope with the challenges they experienced if certain conditions were met.

**Conclusions:**

In response to the challenges informal caregivers experienced when seeking support, improvements are needed to better consider informal caregiver needs within healthcare systems, and to develop interventions tailored to informal caregiver needs and context. Within the healthcare system, informal caregivers may benefit from system navigation support and better integration within healthcare teams to ensure their informational support needs are met. New interventions developed to support informal caregivers should fit within their existing support systems and incorporate the qualities of support, such as empathy, that were valued. Additionally, use of an equity framework and user-centered design approaches during intervention development could help ensure interventions are accessible and acceptable.

**Supplementary Information:**

The online version contains supplementary material available at 10.1186/s12882-023-03444-3.

## Introduction

Informal caregivers, hereafter referred to as caregivers, are family and friends who provide unpaid care and support to people living with chronic kidney disease (CKD). Caregivers may support people living with CKD by assisting with tasks such as help around the house, help with diet and lifestyle changes, help with medical care (e.g., managing medications, communicating with healthcare providers, transportation to appointments, help with treatments), and providing emotional support [1–3]. Time spent caring for someone living with CKD can be similar to full-time employment, involving 34 h or more of care each week [4]. CKD is a complex condition due to treatment complexities (e.g., multiple medications, lifestyle interventions, treatments involving diverse groups of healthcare professionals) and the common presence of co-morbid conditions (e.g., diabetes cardiovascular disease) which can make caring for someone with CKD particularly challenging [5–8].

Caregivings commonly report high levels of burden, and symptoms of mental health difficulties, such as anxiety and depression [4, 9–11]. Levels of burden and mental health difficulties among caregivers are influenced by factors including caregiver and care recipient sociodemographics (e.g., age, income), the care recipient’s health, and social support [12, 13]. Lack of social support for caregivers of people living with CKD is associated with burden, stress, anxiety, and depression [12–14]. Understanding caregivers’ current experiences of accessing and receiving support may help identify areas where support could be improved, which could enhance their well-being.

Studies exploring the general experience of caring for someone living with CKD have commonly identified a lack of support for caregivers, both from social networks (i.e., family and friends) and health and social care professionals [3, 15–18]. However, few studies have explored the experiences these caregivers have accessing and receiving support in more depth. Experiences of support have received greater attention amongst other groups of caregivers, such as caregivers of people living with cancer or dementia [19–26]. Amongst other caregiver groups, caregiving resulted in reductions in social network members for reasons such as lack of understanding of the caregiving situation, or caregivers purposefully distancing themselves from more burdensome social connections [19, 22, 26]. In contrast, some caregivers formed new relationships with people supporting them, including healthcare professionals and other caregivers [19, 23]. The strength of existing relationships may also change over the course of the caregiving experience, impacting support availability [19]. Exploration of similar dimensions of support (e.g., sources of support, perceptions of support) among caregivers of people living with CKD could provide valuable insights into gaps in caregiver support networks.

Understanding caregivers’ experiences of support, such as sources of support and areas where support needs are currently unmet, can provide important information about the wider context in which caregivers provide care [27]. Within the Medical Research Council framework for the development of complex interventions, understanding context is an important element of intervention development, potentially facilitating better integration of new interventions within existing support systems [28, 29]. Context is a multi-faceted concept related to the setting or situation in which an intervention is implemented, such as characteristics of organisations implementing the intervention (e.g., organisational culture) and intervention recipients (e.g., needs, resources, social networks) [27]. To develop a better understanding of the context of caregivers of people living with CKD in the UK, caregiver intervention needs and preferences [1] and stakeholder views of interventions for caregivers [30] have been explored in previous studies. The overall goal of the present study was to examine another dimension of context, namely caregiver’s existing systems of support. The aim was to explore caregivers’ experiences of accessing and receiving support whilst caring for someone living with CKD.

## Methods

### Study design

Semi-structured interviews and reflexive thematic analysis [31] were used to explore caregivers’ experiences of accessing and receiving support. Results are reported following the Standards for Reporting Qualitative Research checklist [32] (Additional file 1).

### Sampling

Caregivers were recruited via online adverts shared via CKD/caregiver specific community organisations (e.g., social media, websites, newsletters), study social media pages, paid social media adverts, and expressions of interest from caregivers participating in a related study exploring intervention preferences [1]. Convenience sampling was used due to resource limitations. Interested caregivers contacted the research team via telephone or email, and were provided with a study information sheet and consent form via email, with the option to receive paper copies by post if needed. Eligible caregivers were adults (18 years of age or older) living in the UK, and currently caring for an adult living with CKD.

### Data collection

Semi-structured interviews, recorded on an external audio recorder, were conducted by CC via telephone (n = 1) or Microsoft Teams (n = 12) between May 2022 and June 2023. Thirteen interviews were conducted, ranging from 43 to 87 min, with a mean length of 63 min. The interview guide explored topics including support needs, sources of support, and barriers and facilitators to accessing support (Additional file 2), and was partly informed by related qualitative research focused on support access and sources of support [19, 33, 34]. Support referred to emotional, practical, and informational support [35]. Interviews were conducted until data was considered as achieving a richness that would tell a multi-faceted story about caregivers’ experiences of accessing and receiving support and was discussed during data collection with JW [36]. No caregivers who took part in an interview withdrew their consent.

### Sample characteristics

All caregivers (n = 13) were female, living in England (n = 12) or Wales (n = 1), with a mean age of 52 (SD = 13). Caregivers had a White British (n = 11) or South Asian (n = 2) ethnic background. Most caregivers (n = 7) had a bachelor’s degree or higher, and were working full- (n = 4) or part-time (n = 3). The majority were caring for their spouse/partner (n = 8) who had been diagnosed with CKD between 2 and 43 years ago. Caregivers had been providing care for a mean of 7.2 years (SD = 7.6) and helped the care recipient with a mean of 10 care-related activities (SD = 6). Activities included providing emotional support (n = 13), managing symptoms (n = 11), running errands (n = 11), and cooking (n = 10). Many caregivers were caring for people with additional co-morbidities including agoraphobia, autism, dementia, diabetes, health-related anxiety, osteoporosis/bone fractures, and polycystic liver disease. Regarding how caregivers were coping with providing care, approximately half felt they were coping neither well nor not well (n = 6), with the remaining either coping well/very well (n = 4), or not well/very unwell (n = 3). Individual caregiver characteristics are presented in Table 1 with pseudonyms.

**Table 1:** Sample characteristics (n = 13)

Name	Age	Relationship to the care recipient(s)	Currently living with care recipient	Care recipient receiving renal replacement therapy?	Length of time caring	How well they were coping
Anna	60–69	Spouse/Partner	No^b^	Yes, dialysis	6 years	Neither well nor not well
Chloe	40–49	Spouse/Partner	Yes	Yes, received transplant	11 years	Neither well nor not well
Claire	60–69	Spouse/Partner	Yes	No^d^	9 years	Very well
Emily	60–69	Spouse/Partner	Yes	Yes, dialysis^d^	1 year 7 months	Not well
Freya	30–39	Spouse/Partner	Yes	No	5 years	Well
Kate	40–49	Spouse/Partner & Parent^a^	Yes	No	2 years 6 months	Very well
Olivia	40–49	Spouse/Partner	Yes	Yes, received transplant	4 years 6 months	Neither well nor not well
Sarah	40–49	Spouse/Partner	Yes	No	3 months	Very unwell
Priya	30–39	Sibling^a^	No	Yes, received transplant	30 years	Well
Rebecca	60–69	Sibling	No	No	10 years 6 months	Neither well nor not well
Sofia	40–49	Sibling	No^c^	Yes, dialysis	5 years	Not well
Holly	60–69	Child	Yes	No	5 years 6 months	Neither well nor not well
Zainab	50–59	Parent	No	Yes, received transplant	2 years 9 months	Neither well nor not well

Note: Pseudonyms and age ranges are used to protect participant confidentiality

^a^Kate and Priya both care for two people living with CKD

^b^Anna’s spouse typically lived with her, however, at the time of the interview he was hospitalised

^c^Sofia provided long-distance care for her brother who lives in another country

^d^Claire and Emily were caring for someone with CKD related to having kidney cancer

### Data processing

Identification numbers were used to link caregivers’ personal information to audio files and interview transcripts. Audio files were transcribed verbatim by a professional transcription company. Interview transcripts were de-identified by the research team by removing identifying information (e.g., names of people and places) before beginning analysis.

### Data analysis

Reflexive thematic analysis [31, 37] was used to the analyse interviews. Given the focus on the subjective, lived experiences of caregivers, thematic analysis was approached using a critical realist perspective [38], with data analysed by primarily considering semantic meanings. CC led the analysis, first reading transcribed interviews to facilitate data familiarisation, with initial ideas and impressions recorded in memos. Interviews were inductively coded, with NVivo used to support data management. Shared meanings across codes were sought to generate initial themes and sub-themes across the whole dataset.

The initial thematic map was discussed with JW for feedback, such as reflecting on the central organising concept of themes. Themes and sub-themes were then revised before being applied to the transcripts to reflect on how well the themes captured the data. Descriptions of themes were developed, with themes and sub-themes continuing to be refined throughout the process. The resulting thematic map, descriptions of themes and sub-themes, and supporting quotes were reviewed by JW and refined. Finally, the revised thematic map and theme descriptions underwent peer examination by PF to facilitate development of the final thematic map. To increase transparency and illustrate how the analysis evolved, thematic maps generated throughout the analysis process can be found in Additional file 3. To enhance rigour, triangulation was undertaken through dialogue with JW, peer examination by PF, with memos taken throughout analysis to maintain an audit trail [39]. Furthermore, disconfirming cases were actively sought [40], however none were identified.

### Researcher characteristics

Interviews and data analysis were conducted by CC who is a white woman, born in Canada and living in Sweden. CC has studied public health and is a PhD candidate with experience conducting research related to caregiver needs and their experiences using e-mental health interventions. JW is a white woman of English heritage, born in the UK and living in Sweden. JW has a PhD in Psychology, is the doctoral supervisor of CC, and has experience conducting, teaching, and supervising qualitative research. JW’s main research interests are in the area of informal care. PF is a white man aged over 50, was raised in Australia, and undertakes clinical practice in a physical health setting with research interests related to cognitive behavioural therapy. Additionally, PF has lived with CKD for over 25 years during which he has experienced all forms of renal replacement therapy and is currently on haemodialysis. There are no pre-existing relationships between the research team and participants.

## Results

Three themes were generated from the data: (1) “Systems seem to get in the way” – challenges within support systems; (2) Relying on yourself; and (3) Support systems can “take the pressure off”. A number of sub-themes were developed within the three themes (Table 2).

**Table 2:** Thematic structure

Theme	Summary	Sub-theme
“Systems seem to get in the way” – challenges within support systems	Caregivers had to navigate complex health and social care systems while seeking and obtaining formal and informal support which presented a number of challenges for caregivers	• “Pushed from pillar to post” - finding your way through health and social care systems • Changing social networks • Systems don’t meet our needs • People don’t understand CKD
Relying on yourself	Caregivers had to identify and access support independently to meet their needs. However, such self-reliance may leave support access barriers unaddressed	• Leveraging existing skills and networks • “We can’t just go out and find it” • When beliefs get in the way
Support systems can “take the pressure off”	Support systems had the ability to reduce the stress and burden caregivers were experiencing if they possessed certain qualities	• Empathetic support that “instinctively knows” what you need • Support you can count on

### Theme 1: “Systems seem to get in the way” – challenges within support systems

Caregiver support systems were both formal (i.e., health and social care system), and informal (i.e., friends and family). This theme describes the challenges caregivers experienced when navigating support systems which could be complex and leave support needs unmet.

“Pushed from pillar to post” - finding your way through health and social care systems: To receive the support and information both caregivers and care recipients needed, caregivers had to navigate through health and social care systems with complex care pathways where there *“wasn’t a streamlined process or anything”* (Priya), in which caregivers were poorly integrated. The health and social care system was viewed as *“over-stretched”* (Chloe), *“under a lot of strain”* (Claire), and *“in a crisis point”* (Priya). While caregivers were understanding of the challenges healthcare systems were experiencing, this was also recognised as a reason for the lack of available support and a source of burden for caregivers who struggled to remain informed regarding their care recipients’ health.

*“Because sometimes I think, we have a wonderful NHS in this country. But it felt like so many of the emotional problems were caused by the systemic issues. It’s really hard to disentangle that because there were so many systemic issues and what is just how you’re feeling because somebody’s sick, and how much of it [is] just because you can’t [get] anybody to pick up the phone and actually tell you what’s going on.”* Olivia.

The poor integration of caregivers into healthcare systems caused frustration, negatively impacting caregiver views of healthcare systems and relationships with healthcare providers. With healthcare systems focused on patients, caregivers felt they *“really don’t count”* (Kate) and were *“dismissed more than supported”* (Olivia). Healthcare professionals were perceived as making assumptions about caregivers’ ability to cope, and lacked sensitivity regarding the challenges caregivers may be facing. Inadequate responses to caregiver needs, and lack of follow-up when reaching out for support not only left caregivers unsupported, but it also damaged the relationships between caregivers and healthcare professionals.

*“I used to phone [my husband’s nurse when I was struggling emotionally]. I’d leave messages, ‘please can you phone me’. […] I was in a right, a real state at the time. ‘Please’, and I’m crying - ‘I don’t know what to do.’ and ‘what can I do?’ And nothing never, ever came back. So that was [when] my estimation of them went completely downhill.”* Emily.

The complexity of structures within healthcare systems was perceived as another reason support for caregivers was lacking. These structures placed distance between caregivers and healthcare professionals, making healthcare professionals hard to access, and causing caregivers to be excluded from communications.

*“Well the hospital and the consultants. Again, that was quite frustrating because systems seem to get in the way. In that they were sending me information and they were sending me the appointment times and then all of a sudden it seemed to be that their system could only send out two letters, one to the main carers, which is the care home, and [one] to the GP, which completely cut out me.”* Rebecca.

In response to the challenges faced within healthcare systems, caregivers adapted their support seeking behaviours. For example, some recognised the need for efficiency during healthcare appointments, leading caregivers to ask their question *“very quickly”* (Zainab), *“before they’re ushering you out the door”* (Freya) while trying not to *“add to people’s workloads”* (Chloe). Some felt forced to make decisions without getting the medical advice they desired, with one caregiver seeking private care for her husband: *“I feel quite sad that we sort of went private for quite a few of these appointments and that’s when we actually got some help”* (Claire). Caregivers also limited the number of times they reached out for support, anticipating difficulties receiving support that was needed, with some caregivers not seeking support at all.

*“And going through the NHS obviously, we all know the NHS at the moment is - the waiting list is forever. So I personally myself haven’t [reached out for support]”* Emily.

Changing social networks: Caregivers experienced changes within their social networks as a result of their caregiving role, impacting available support. Caregivers experienced losses within their social network due to a lack of sensitivity from some network members regarding the impact of caregiving. Network members were perceived as distancing themselves from caregivers for reasons such as feeling uncomfortable discussing the care recipient’s situation, *“some people, I think, are still really awkward talking about medical stuff”* (Freya), or due to caregivers not being available to meet or take part in social activities, *“you kind of keep saying no, or you aren’t able to […] do some things, and then people just stop asking”* (Chloe). Network members were not always willing to adjust the dynamic of their relationship with the caregiver to adapt to the caregivers’ needs.

*“Just that because you haven’t got the freedom, then they [family and friends] don’t necessarily understand why you haven’t got that freedom anymore and maybe misinterpret the fact that you’re not around as much […] So as an example, we’ve got a very close friend who lives [a] two and a half hour’s drive away and we can’t just say, ‘let’s meet up tomorrow and have a lunch’ or something like that. You can’t do it because you’ve got to make sure that there’s someone here for mum. […] So, it’s difficult to arrange and people don’t always want to plan three or four weeks ahead.”* Holly.

Caregivers also purposefully reduced contact with their social networks in response to caring for someone with CKD. Some caregivers actively chose to reduce contact with network members to spend more time with their care recipient or to distance themselves from more burdensome ties. For Zainab, who has a South Asian background, cultural beliefs about the causes of physical health conditions caused her social network to change as she distanced herself from some family members who would not be supportive of her or her son.

*“Then there was the Asian dynamic as well. Some Asian communities are quite backwards. So my son didn’t want to tell some of the family, because they’re quite mediaeval in the thinking. So, they would have said, […] I’ve done something bad, I’ve sinned, that’s what’s happened, so the result is, I’ve been punished because something’s happened to him. So, he said, ‘I don’t want them to know’. So, for nearly 20 months I reduced contact with wider family on my side, on the Asian family side, because I just couldn’t manage that dynamic.”* Zainab.

In some cases, reduced network contact was not the choice of the caregiver. For example, Chloe had to limit contacts due to living with someone who is immunosuppressed during the COVID-19 pandemic which *“reduced the amount of help that I could get from them [family]”*.

Systems don’t meet our needs: Caregivers recognised gaps in available support which left support needs unmet. Regardless of the care recipient’s CKD stage or treatment, caregivers felt basic information about CKD and the impact it can have on the care recipient’s physical health and relationships was lacking. This impacted their ability to provide care and support to their care recipient.

*“I think what would have been good from my point of view was for them to give us some information on why this [kidney failure] has happened. What the stages are. We didn’t even know what the stages were they just said ‘you’re stage five kidney failure’. And I didn’t know if that was the first stage or the last stage. It was really, really confusing.”* Emily.

Given how complex a CKD diagnosis can be in relation to different treatment types (e.g., medication and monitoring only, dialysis, transplant) and CKD stages, some caregivers recognised gaps were linked to support being unable to meet their needs throughout different phases of the CKD journey. Claire, whose spouse decided he did not want to receive dialysis, felt support was withdrawn once renal replacement therapy was no longer being considered. For caregivers of people receiving a transplant or on the waiting list for a transplant, there were a number of stages during the transplant journey where support was felt to be missing, such as after kidney donation, when potential transplant opportunities did not move forward, or when waiting for a transplant:

*“You go through that process, you get told that you’re on the transplant list, but then that’s it. Because you are just waiting for that very special call. And I think that’s perhaps where, from a kidney and a liver point of view, that there just is maybe no support for the patients, [or] the families.”* Kate.

Support that was sensitive to the cultural background of caregiver’s was important to Priya and Zainab. Both experienced being unsatisfied with support received from people who did not share their cultural background and did not understand how culture impacted their caregiving experiences. Support that was not culturally sensitive posed a burden as they did not want to feel they were *“spending hours just trying to explain my culture to somebody”* (Priya).

People don’t understand CKD: Understanding of CKD was lacking among caregiver social networks and non-renal healthcare professionals and organisations. This created unsatisfactory experiences with support. Non-renal healthcare professionals’ poor understanding of CKD led to negative experiences receiving support. For example, when Sofia accessed mental health counselling via her workplace, the counsellor *“didn’t even know what dialysis was. So, it was irritating”*. Without network members and support providers having an understanding of CKD, support was not perceived as helpful.

*“[…] it’s a very isolated experience because not everyone fully understands. I think that’s just one of the things with CKD, not everyone has an understanding of what it is and what it entails. So people might make recommendations that are just not helpful. It’s not because they’re being ignorant, they’re just trying to help, but actually it’s not very helpful.”* Priya.

This also meant caregivers had to explain their behaviour and decisions to others. For example, Chloe was frustrated she had to justify the need to maintain some COVID-19 precautions due to living with someone who is immunosuppressed. Claire, Emily, and Kate recognised a contrast between the understanding of CKD among their social network compared to other conditions such as cancer or heart attacks, which attracted more support and sympathy.

*“And I think for my journey if more people knew about [my husband]’s disease, I wouldn’t find that I’d have to explain so much and take so much of my time and effort into justifying what’s wrong. Whereas if somebody says to you, ‘Oh I’ve got cancer’ you immediately think, ‘Oh wow.’ Whereas with [my husband it’s], ‘Well what’s that?’ ‘What does that mean?’ And you just think, oh my life, this is exhausting. So the more that the general public know about the disease, the easier it is for people like me.”* Kate.

Perceptions that existing support was not tailored enough to the context of caring for someone with CKD deterred some caregivers from seeking support as they were unsure how suitable the support would be. Generic caregiver support was perceived by some as burdensome and caregivers did not “*want to waste time*” (Zainab) interacting with organisations that “*don’t really understand renal issues*” (Zainab).

### Theme 2: relying on yourself

In response to support systems not meeting caregiver needs (Theme 1), this theme describes how caregivers expressed the need to be resourceful and rely on themselves to find and access support. However, this approach to obtaining support left support access barriers unaddressed.

Leveraging existing skills and networks: Caregivers had to use their own skills and initiative to learn about CKD, available support, and implement strategies to support their own well-being. Information was often obtained independently by searching for information online (e.g., websites, Facebook groups), identifying community organisations and requesting information, obtaining books, and engaging their wider social network. Caregivers leveraged their own skills to find information to meet their needs, facilitating support access, such as Priya who has a background in research: *“I know where to look for information, I’m able to research things”*. Independent information seeking was a necessary activity given healthcare teams did not always provide adequate information.

*“We went to the first appointment and the doctor said, ‘right yes, you’ve got PKD [polycystic kidney disease], you’re going to need a kidney transplant at some point and your kids might get it too, bye’. I was like, ‘what?’ It was me who went home and did all the research and joined all the groups, did all the reading. […] So, I did all of the research and looked into a lot more than we received.”* Freya.

The internet was a common and valuable tool to find support, however, caregivers expressed the need for caution when searching for information online as information was viewed as possibly being *“not reliable”* (Priya) or increasing anxieties.

*“I’m not a real Google fan because I think you Google things and you think, oh my God”* Emily.

Caregivers’ engaged their wider networks to obtain medical information from social network members with medical training such as retired midwives and nurses, a paramedic and an anaesthesiologist. These social networks helped fill gaps related to understanding CKD, treatments, and symptoms as Zainab shared: *“they [retired midwives and nurses in my writing group] were very good at explaining smaller things that I didn’t understand”*. Priya resorted to seeking medical advice from healthcare professionals with expertise in certain treatment options on LinkedIn as she was not receiving the support needed from her sister’s healthcare team.

*“I’ve stalked consultants on LinkedIn and then messaged them ‘my sister’s going through this, and we just wanted your advice, seeing that you’ve done research on this […] can you advise us on this?’ One person was really helpful and she totally ghosted me afterwards, but that was obviously really weird that somebody was just messaging them on LinkedIn. But I was so desperate at that point. Which is saying something, isn’t it? […] At that moment in time, I was so desperate that I was relying on somebody on LinkedIn and that’s the desperation that I felt. That’s pretty bad, isn’t it?”* Priya.

Finally, caregivers also used their own self-care skills to maintain their well-being, spending time on activities they enjoyed such as physical activity and creative arts (e.g. writing). Freya, Kate, Priya, and Sofia were also motivated to become involved with CKD awareness and research.

*“It’s [a writing group], not a direct support [or] a specialist group, but I found that to be really good because I felt I needed to keep some of my normal things, everyday things going, because otherwise you lose yourself and you get angry with it and you lash out. You think, well, my life’s just become a carer now and I’m just worrying all the time, it’s all negative. So I made myself do my sessions and my writing and things just to help me, all my activities to keep me positive, so I could put things into perspective.”* Zainab.

“We can’t just go out and find it”: Caregivers perceived barriers related to their lack of knowledge about available support, the caregiving role, and competing demands which made accessing support challenging. Caregivers lacked knowledge and information about where they could obtain support, making seeking support *“really frustrating”* (Holly) and forced caregivers to invest a significant amount of time researching available support. However, caregivers seldom had time to find support given they were managing caregiving responsibilities, and other responsibilities such as childcare and employment. The unpredictability of their caring situation also made it challenging to commit to support programmes available.

*“You put your own needs on the back burner because you have to deal with what’s urgent and what’s required of [you] when you’re caring for your family member.”* (Priya).

The significant individual burden to find and obtain support was viewed as problematic. Caregiver’s ability to seek support could be negatively impacted when experiencing emotional distress: *“when we’re depressed it’s even harder to be self-motivated”* (Olivia). Additionally, not all caregivers were expected to have the skills and/or resources to navigate complex and unfamiliar systems to find the support they need as Anna shared: *“I don’t know what happens to people who aren’t capable”*.

When beliefs get in the way: Negative beliefs regarding support programs, and feeling undeserving and guilty for seeking support, had to be overcome for caregivers to access support. Some caregivers viewed mental health support as emotionally challenging, causing caregivers to relive negative experiences and bringing up negative emotions, with Chloe feeling she lacked capacity to engage with mental health support: *“I’m just not willing to go through the pain”*. For some, accessing support caused anxiety and worry that it could have a negative impact on them.

*“I was mindful that I didn’t know that I could handle other people’s desperate stories if I had my own”* Olivia.

Caregivers struggled with the belief that they were less deserving of support than others, leading to feelings of guilt surrounding support seeking. Caregivers felt they should be able to manage their caregiving responsibilities without needing support and were anxious about being perceived as unable to cope. Caregivers struggled to justify their need for support, often comparing their needs with the hypothetical support needs of others who were assumed to be in greater need of support, in particular comparing their needs to those of their care recipient or others living with CKD: *“I wouldn’t like to derive resources from people that are actual kidney patients that may need that support 10 times more than I do”* (Sofia). This caused caregivers to question whether available support was intended for them and whether they were deserving of support.

*“Feeling like it’s not critical for me to get this, to access this because I think that’s rooted in the guilt that I feel because I’m technically healthy or normal. I don’t like using that word, but it’s often the word that gets used. Because I don’t have [any] health conditions, I feel like I shouldn’t need this support. So that would stop me in the past and still does to an extent.”* Priya.

### Theme 3: support systems can “take the pressure off”

This theme reflects how support systems could provide caregivers with reassurance and relief while they were coping with caregiving related challenges if systems possessed key qualities that made support appropriate. Although support systems perceived as empathetic and reliable helped alleviate caregiver burden, they did not necessarily negate the challenges caregivers experienced when navigating health and social care systems (Theme 1).

Empathetic support that “instinctively knows” what you need: Support systems alleviated some burdens experienced by providing empathetic support. Empathy from formal and informal network members enhanced the support provided given empathetic network members were better able to understand when and what kind of support was needed. Caregiver’s received empathetic support from a variety of people such as family and friends, neighbours, work colleagues, healthcare professionals, and other caregivers. Support from network members with lived experiences relating to the caregivers’ situation, such as having medical knowledge, or having experience caring for someone with CKD or any other condition, was especially valued. Support from other caregivers was also appreciated given it opened the possibility for support to be reciprocated.

*“So, [we] are in touch with another couple that have been on the same journey as us […] And we have become great friends. And we find that we can send each other a text as a couple or as a female to a female and ask about our husbands. But we actually take the time to ask about each other. And it means a little bit more because if one of us replies, ‘I’m actually having a really tough day today’, whereas somebody [else] will go, ‘oh it’s okay, tomorrow will be better’, they will instinctively know what it could be and will ask the right questions.”* Kate.

Empathy was shown when network members demonstrated they understood caregiver needs such as employers providing flexible working hours to accommodate caregiving responsibilities, or friends and family taking action to learn about CKD. However, what was viewed as empathetic was subjective. For example, Sofia valued when network members asked about her brother as she felt they understood how important her brother is to her. Conversely, Claire did not always appreciate when network members asked about her husband as she felt her own well-being and the impact caregiving was having on her was ignored.

*“Some friends, they want to know every day an update or something. You just [think], oh for goodness sake, just don’t worry about [it], […] it’s not, ‘well how areyoudoing?’”* (Claire, emphasis added).

Support you can count on: The reliability of formal and informal support networks was valued as it provided caregivers with confidence that support would be available when needed. Reliability was often the result of effective communication, and strong relationships with network members. Caregivers valued support that was easy to access and when network members quickly responded to support requests. Among formal support networks (i.e., healthcare professionals), having a consistent healthcare team or contact point, and receiving information efficiently provided reassurance to caregivers and built trust. Informal support networks (i.e., family, friends, neighbours) also provided reliable support which caregivers felt they could rely on, including in emergencies when support needs were unanticipated.

*“Our neighbours where we live, there’s only nine people [who] live here. We’re all spread out because obviously [it’s] quite rural. But we all became quite close during lockdown because we started a WhatsApp group […] And they all know [my husband has CKD] […] which is really nice, as we’re always the first port of call, ‘Can I get you anything? Do you need anything?’ And they’re always asking […] So I feel that if something really desperately terrible happened or whatever I would have the support here, definitely.”* Emily.

## Discussion

By exploring caregivers’ experiences of support, key characteristics of current support systems were identified which highlight the need to improve caregiver support provision. Support systems could be complex and challenging to navigate, leaving caregivers with unmet needs and feeling dissatisfied with available support. Given support systems did not meet all caregiver needs, caregivers relied on their own abilities and social networks to ensure their needs were met. However, this could negatively impact support access as caregivers were left to cope with support access barriers independently. Support systems also had the ability to provide caregivers with support which helped caregivers cope with the challenges of providing care if support was perceived as reliable and empathetic.

Many challenges caregivers encountered related to navigating unfamiliar and disjointed health and social care systems where communication challenges made it difficult for caregivers to receive adequate support, especially informational support from healthcare professionals. Unmet information needs among caregivers have been reported in other studies, suggesting caregivers do not receive adequate CKD information, and are excluded from some conversations with healthcare professionals [15, 19, 41, 42]. One factor which may contribute to the unmet information needs among caregivers is the complexity within CKD care regarding CKD stages and treatment options, and presence of comorbidities (e.g., diabetes, hypertension) [6, 43]. Given the common presence of comorbidities, people living with CKD and their caregivers interact with a wide range of healthcare professionals who may not have renal expertise [6, 44]. Non-renal healthcare professionals may not have the educational resources available to provide people living with CKD and their caregivers with adequate information [44, 45]. This potentially contributes to the unmet needs and unsatisfactory experiences with support reported in this study.

Many of the challenges caregivers encountered when navigating health and social care systems illustrated how poorly caregivers are integrated into clinical practice. Integration of caregivers into healthcare systems is a common challenge, with healthcare professionals experiencing challenges integrating caregivers into their practice, and the absence of a standardised approach to provide caregivers with information [42, 46–48]. Renal healthcare professionals have recognised challenges communicating with caregivers [49, 50], with one study showing dialysis nurses were aware caregivers may not be satisfied with information provided [50]. As a result of inadequate inclusion of caregivers within healthcare systems, caregivers have relied on informal information sources (e.g., internet, peers) or obtained information indirectly by observing healthcare professionals [51, 52]. Policy and practice changes are needed to ensure caregivers are consistently included in communications regarding the care recipient’s care, information needs are addressed, and system navigation is supported.

Reliable support which was empathetic regarding the caregiving experience and impact of CKD was valued. Qualities caregivers appreciated when receiving support were not necessarily related to the role of the network member. For example, caregivers received empathy and understanding from a variety of network members such as friends, family, healthcare professionals, and other caregivers. Empathy has been identified as an important dimension of support, both within competency frameworks for mental healthcare professionals [53–55], and literature focused on intervention development for caregivers [19, 56, 57]. By focusing on support characteristics valued by caregivers, it may be possible to enhance intervention acceptability by ensuring support interventions embody the characteristics caregivers value within their existing support systems.

Caregivers reported relying on their own skills and resources to find and access support, a finding which has also been reported elsewhere [42, 52]. However, self-reliance to find and access support raises equity concerns. Inequitable access to physical and mental healthcare based on characteristics such as socioeconomic status and ethnicity is well established among the general population, and can be linked to variation in individuals’ abilities to seek and access healthcare services [58–62]. Similar equity concerns have been raised regarding support interventions for caregivers given certain dimensions of equity (e.g., culture, disability, religion, gender) are not often considered when interventions are developed and implemented [63, 64]. Indeed, this study showed that caregivers with South Asian backgrounds felt culturally appropriate support was lacking. In addition, evidence suggests higher levels of burden and unmet needs among caregivers are associated with lower socioeconomic status and ethnic background [65–67]. To develop interventions that facilitate equitable support access, all dimensions of equity frameworks [68, 69] should be considered during intervention development.

### Implications for intervention development

Existing caregiver support appeared to leave support needs unaddressed, therefore, the development of support interventions to address caregivers’ informational and emotional support needs may be beneficial. Exploring caregivers’ experiences of receiving and accessing support contributes to the consideration of context within the intervention development phase of the Medical Research Council framework for the development of complex interventions [29]. Findings of this work, supported by additional research [1, 30], can be used to suggest recommendations for future intervention development.

Caregivers reported receiving support from a variety of sources (e.g., healthcare professionals, community organisations, colleagues, friends, family, neighbours), however, sources of support varied individually. For example, not all caregivers received much support or had a strong relationship with the care recipient’s healthcare team, and not all caregivers interacted with community organisations to the same extent. Given the composition of caregiver social networks varied, implementation of interventions for caregivers could be enhanced by creating multi-sectoral intervention referral pathways that support referral through both healthcare systems and community organisations. Social networks of caregivers and professionals should be explored in more depth using social network analysis methods to gain a more detailed understanding of where caregivers receive support to identify additional potential referral pathways and identify key professionals to involve during intervention implementation [70].

Navigating health and social care systems was challenging for caregivers. Caregivers often interacted with different sectors of healthcare systems (e.g., renal team, GP, teams supporting treatment of comorbidities) and non-renal healthcare professionals could have limited understanding of CKD [44, 45]. To help caregivers navigate health and social care systems, system navigation support could be incorporated into interventions [71, 72]. Existing roles, such as Renal Assistant Wellbeing Practitioners, could be leveraged to provide caregivers with support navigating healthcare systems and support implementation of interventions for caregivers through intervention endorsement and/or delivery [53]. Interventions for caregivers could also include content related to communication with healthcare providers and understanding the healthcare system to provide caregivers with needed knowledge and skills to improve discussions with healthcare professionals [73, 74].

Perceiving support as empathetic and understanding was an important factor for caregivers to view their experiences with support positively, highlighting the importance of building empathy into interventions. Involving healthcare professionals and staff from community organisations who have their own experiences providing informal care during intervention delivery could enhance caregivers’ perceptions of empathy when using interventions [30]. Another strategy to enhance empathetic interactions within the healthcare system, and ensure interventions for caregivers embody empathy is to provide health and social care professionals with communication training. Communication training has been shown to improve health and social care professionals’ ability to demonstrate empathy and improve conversations with patients and caregivers (e.g., asking open questions, eliciting caregiver’s concerns) [75, 76]. Given demonstrating empathy does not rely on renal specific knowledge, communication training could benefit renal and non-renal health and social care professionals working with caregivers, or professionals involved in intervention delivery.

Interventions can also be designed to provide caregivers with empathy and understanding by tailoring interventions to caregiver characteristics (e.g., cultural background) and caring situations (e.g., stage of care recipient’s CKD, treatment modality) to develop content that reflects caregivers’ lived experiences [57]. Tailoring has been found to enhance the relevance and acceptability of interventions for caregivers of people living with CKD and caregivers of people living with other health conditions [1, 77–81]. Additionally, tailoring interventions based on caregiver’s cultural background may enhance intervention effectiveness [82, 83], and has been found to improve the acceptability of interventions for caregivers in other settings [84]. Consideration of culture is also one dimension of equity which, as discussed above, requires further consideration during intervention development for caregivers [63, 64, 68, 69]. Use of public contribution, engagement of caregivers with different cultural backgrounds during intervention development, and user-centred design approaches (e.g., creating personas) could be strategies to enhance acceptability and build empathy into interventions for caregivers [85–87].

### Limitations

Caregivers of people living with any stage of CKD and receiving any treatment type could participate in this study, reflecting the diverse caregiving situations within CKD care. However, given caregivers reported different levels of support based on the treatment their care recipient was receiving, it may be important to further explore needs within sub-groups of CKD caregivers (e.g., caregivers of people receiving dialysis, caregivers of people living with a transplant, caregivers of children and young adults). All caregivers were women, therefore, the experiences men have receiving and accessing support were not necessarily captured within this study, limiting the transferability of findings. This may be important when interpreting results given gender can impact experiences of distress [88] and men may have different support seeking behaviours [89–93], which could influence experiences of accessing and receiving support. Two participants had a South Asian ethnic background and they shared that some experiences with support were influenced by their cultural background. Future work should focus on further exploring the experiences caregivers with diverse ethnic backgrounds have receiving and accessing support to facilitate development of equity focused interventions. This study primarily relied on recruitment via social media and community organisations, however, using additional recruitment methods, such as recruitment via healthcare services, could be a strategy to reach more diverse groups of caregivers. Finally, public and stakeholder involvement was not conducted as part of this study. Involving caregivers and/or kidney healthcare professionals throughout the design and conduct of this study may have enhanced recruitment, and provided another important perspective on the results.

## Conclusions

Caregivers of people living with CKD had both positive and negative experiences with support received from a wide variety of sources including family, friends, neighbours, colleagues, community organisations, healthcare professionals, peers, and online resources. Caregivers experienced many challenges navigating systems which had complex structures, did not meet their needs, and were poorly interconnected. However, caregivers also experienced support which was empathetic and reliable, which helped caregivers cope with the challenges they experienced as a result of the caregiving role. To fill gaps left in their support systems, caregivers used their own skills to find support, which may lead to inequities in relation to the support received.

To better address caregiver support needs, existing practice should better integrate caregivers within the healthcare system to ensure their needs are considered by healthcare professionals, and new support interventions should be developed that are tailored to caregivers needs and context. Interventions that are well integrated into caregivers’ current support systems (e.g., diverse multi-sector information and referral pathways), and encompass support characteristics valued by caregivers (e.g., empathy) may be more acceptable and have greater implementation potential. Future research should further explore the needs among specific sub-groups of caregivers of people living with CKD to ensure interventions are adequately tailored.

### Electronic supplementary material

Below is the link to the electronic supplementary material.

Supplementary Material 1

Supplementary Material 2

Supplementary Material 3

Supplementary Material 4

## Data Availability

The datasets generated and/or analysed during the current study are not publicly available to maintain participant’s privacy and confidentiality, but may be provided upon reasonable request from Dr. Joanne Woodford (joanne.woodford@kbh.uu.se).

## Abbreviations

CKD: Chronic Kidney Disease
UK: United Kingdom
