# Supplementary material for: “Systems seem to get in the way”: a qualitative study exploring experiences of accessing and receiving support among informal caregivers of people living with chronic kidney disease
Source: BMC Nephrol. 2024 Jan 3;25:7. doi: 10.1186/s12882-023-03444-3 (PMC10765659; doi:10.1186/s12882-023-03444-3)
Supplement: Supplementary file 2 — Supplementary Material 2 [file 12882_2023_3444_MOESM2_ESM.pdf]

Interview Guide for Caregivers

Introduction

Welcome to the interview [Important: ensure participants are reminded of the purpose of the interview and re-affirm consent].

1. Can you tell me a little about your caring situation?
    a. PROMPTS: Who do you care for? What kidney condition do they have? What stage are they at? What type of treatment are they receiving? How long have you cared for them? What type of things do you help them with (e.g. practical support, emotional support, decision making etc)?

Needs

2. What type of support would you like to receive to help you in your caring role?
    a. PROMPTS: Information about [friend, family member, partner's] condition or support resources? Financial support? Practical help with providing care? Help balancing work and providing care? Emotional support?
    b. PROMPT (if response is focused on support received): Is there a type of support that you wish you would have had, but have not received?

Sources of support

3. What type of support have you received from healthcare professionals in relation to your role helping [friend, family member, partner]?
    a. PROMPTS: What were your impressions of these healthcare professionals (e.g. trustworthy/reliable, approachable)? What went well in your interactions with them? What did not go so well in your interactions with them?
    b. What type of support did they provide you with (e.g. information, practical support, emotional support, referral to other resources)? How helpful was the support you received from them?
    c. PROMPT: What support from healthcare professionals have you received in relation to your own emotional wellbeing?

4. What type of support have you received from community organisations or charities (e.g. Kidney Care UK, National Kidney Federation, Kidney Patient Associations, Carers UK) in relation to your role helping [friend, family member, partner]?
    a. PROMPTS: What were your impressions of these community organisations (e.g. trustworthy/reliable, approachable)? What went well in your interactions with them? What did not go so well in your interactions with them?
    b. What type of support did they provide you with (e.g. information, practical support, emotional support, referral to other resources)? How helpful was the support you received from them?
    c. PROMPT: What support from community organisations or charities have you received in relation to your own emotional wellbeing?

5. What type of support have you received from your family, friends, neighbours, and colleagues in relation to your role helping [friend, family member, partner]?
    a. What type of support did they provide you with (e.g. information, practical support, emotional support, referral to other resources)? How helpful was the support you received from them?
    b. PROMPT: What support from family, friends, neighbours, and colleagues have you received in relation to your own emotional wellbeing?
    c. Sometimes when people start helping someone with a chronic kidney condition, they find that their social network, e.g., the network of people you interact with and have relationships with, can change. In what ways has your social network changed since you started helping someone with a kidney condition?
        i. PROMPTS: What happened with the people you already knew (did you see them more or less, how if your relationship with them now)? What new relationships have you made?

Barriers and facilitators to accessing support

6. What factors have made it difficult/stopped you from getting support?
    a. PROMPTS: lack of time, hard to make time for yourself, don't know where to go, uncomfortable talking to people about need for support?

7. What has helped you/made it easier to get support?
    a. PROMPTS: do you know where to go if you need support? Do you feel you have someone you can speak to if you need support? Are you comfortable asking for support?

Views on e-mental health

Next I want to ask you a few questions about online emotional support programmes. As part of this project, we asked you to read through some brief material describing what online emotional support programmes are. [Summarize material before moving to next question].

8. What is your experience of using online programmes such as websites and smartphone applications in connection with your emotional wellbeing?
    a. Can you give me examples of when you have used an online programme in connection with your emotional wellbeing?
    b. If not, what are your thoughts about receiving an emotional support programme using digital solutions?

10. What would an online emotional support programme need to include to meet your needs and preferences?

a. PROMPTS: What topics should be included? Who would you like to support the programme? How would you like to be supported e.g., face-to-face, telephone, video-conference, email? How should the programme be made available (e.g., through the kidney unit, a community organization, general practitioner)?

11. What opportunities or benefits do you see for an online emotional support programme to be successful in supporting your emotional health?

a. PROMPT: What would motivate you to use an online emotional support programme? Helpful in overcoming practical barriers? Stigma. Time.

12. What obstacles do you see for an online emotional support programme to be successful?

a. PROMPTS: Safety, security, trust, burden of use, perceived effectiveness.

13. Is there anything else you would like to share about your experiences accessing support, your needs, or about online emotional support programmes.
