# Supplementary material for: “Systems seem to get in the way”: a qualitative study exploring experiences of accessing and receiving support among informal caregivers of people living with chronic kidney disease
Source: BMC Nephrol. 2024 Jan 3;25:7. doi: 10.1186/s12882-023-03444-3 (PMC10765659; doi:10.1186/s12882-023-03444-3)
Supplement: Supplementary file 3 — Supplementary Material 3 [file 12882_2023_3444_MOESM3_ESM.pdf]

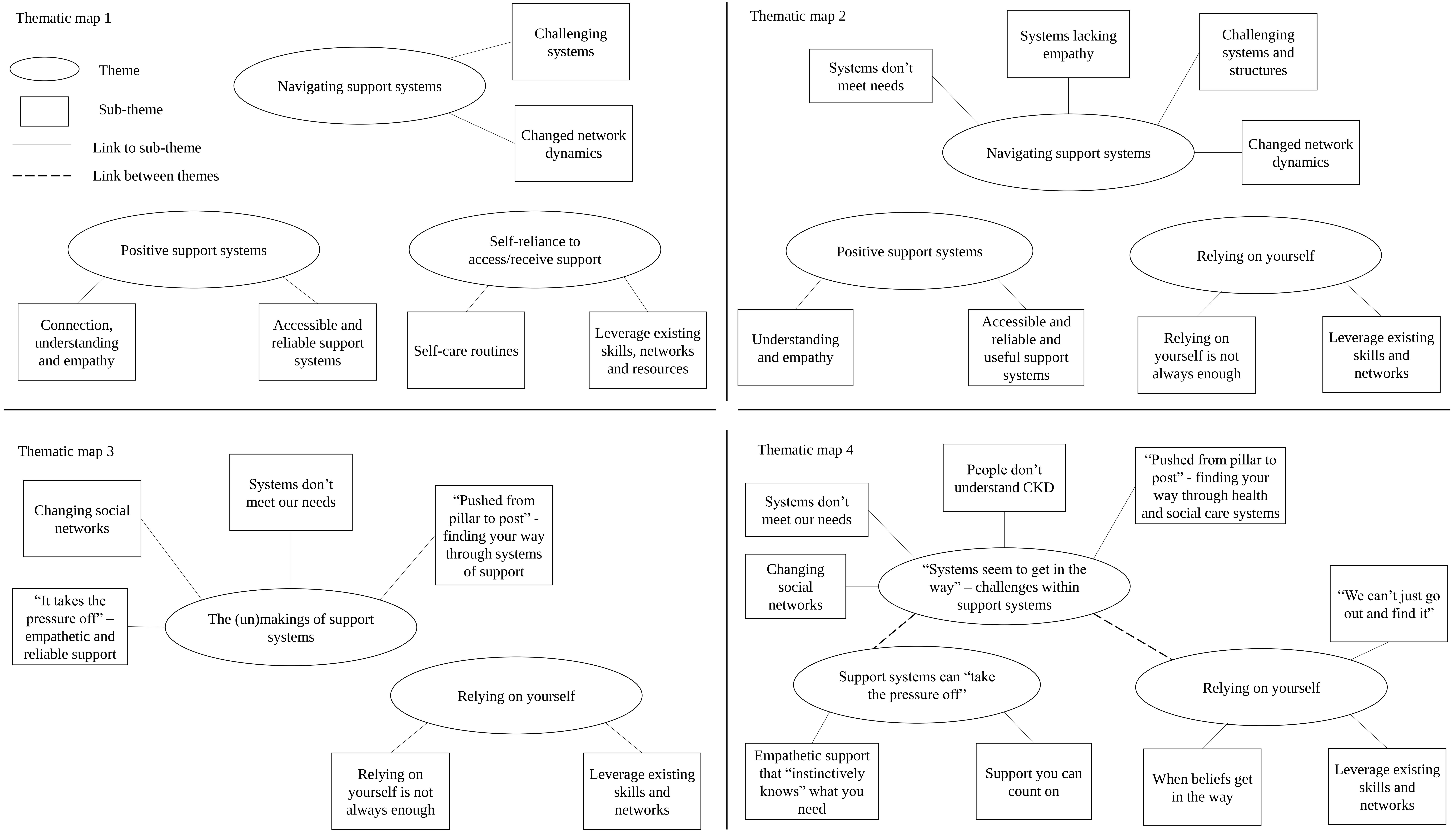

Note: Thematic map 4 is the final version of the thematic map presented within the paper
