# Supplementary material for: “Systems seem to get in the way”: a qualitative study exploring experiences of accessing and receiving support among informal caregivers of people living with chronic kidney disease
Source: BMC Nephrol. 2024 Jan 3;25:7. doi: 10.1186/s12882-023-03444-3 (PMC10765659; doi:10.1186/s12882-023-03444-3)
Supplement: Supplementary file 4 — Supplementary Material 4 [file 12882_2023_3444_MOESM4_ESM.pdf]

Additional file 4: Additional quotes supporting themes and sub-themes

Theme	Sub-theme	Quotes
"Systems seem to get in the way" – challenges within support systems	"Pushed from pillar to post" - finding your way through health and social care systems	*"You know one of the things that's a huge challenge when somebody is as sick as [my husband] was, is just joining up all the bits that you sort of feel should be joined up anyway. And that huge amounts of your time are just spent trying to get one health professional in one place to talk to the other one."* Olivia <br>*"And then the other thing that really annoyed me was the last appointment we went to which is from the renal consultant who he will be seeing every sort of six months, who's based at the Renal Centre. And he sort of said, well yes, I think we should refer you to the hospice team, community team. But the GP has never received a letter about that to request that. And it's only because I wrote to the GP and they know that"* Claire <br>*"I caught it [Covid] and isolated in the house, and my husband phoned his consultant and said what do I do should I catch Covid? But his consultant didn't get back to him. So even to this day, we still are none the wiser […] that was the frustrating bit that, even though we did phone the hospital, we didn't get a response back."* Sarah
	Changing social networks	*"Even though, chronic kidney disease has brought us together as a family, on an emotional level, I'm not close to my parents. I don't turn to my parents if I need something. They're the last people I will turn to because I don't want to burden them with more stuff."* Priya <br>*"From us being in a group of maybe 12 or 14, we can count on one hand the people that are still our friends. […] Some of them just can't cope and deal with the fact that [my husband] is poorly. And that he's deteriorated and that he can't do the things that he did before. So they selfishly*

		don't want to change their lives, which I completely understand. But they also emotionally find it very hard to be with us. And when we were going through the transplant assessment, you talk about your own mortality […]. And they found it extremely hard that we could talk about it. We also had some that we just found that we had nothing in common anymore. So the things that perhaps kept us as friends, the going out for nice meals, we didn't do that, so there was nothing to talk about […] So they sort of diminished."<br>Kate<br><br>*"Family wise it's difficult, I've got an older sister who comes and covers for a couple of times a year so that we can get a longer break away. But our relationship with her has deteriorated really because of it. Because she doesn't believe mum is as ill as she is. So, there's conflict there and my sister sometimes tells us what we're doing wrong when caring for her. […] But we've got different viewpoints, haven't we? We're here every day, all day. We know what works and what doesn't. She comes in for four weeks a year and doesn't see what we see."*<br>Holly
	Systems don't meet our needs	*"All those renal teams said was that he has to be careful. […] What on earth does that mean?"*<br>Chloe<br><br>*"There doesn't seem to be anything for people that have chosen not to go down the treatment route. But that might just be how I've sort of seen it."*<br>Claire<br><br>*"I think there needs to be more support out there for families. There needs to be a safe space where you can say, 'oh, this is what I'm dealing with too'. You don't want to take anything away from the patient, but you need to say, well, I need support too. I think a lot of what's out there that we've seen isn't really designed for families."*<br>Freya

		"Consultants can be very professional and they're busy. They can talk to you very much sometimes in jargon and I'm thinking, 'well what does that mean?' […] So whilst the doctors can say, 'right, well, her blood pressure's this and this is that and we'll see you again in three months', sometimes that's not enough." Rebecca
	People don't understand CKD	*"Generic charities and local authorities don't really understand renal issues. So, I just felt I don't want to go to an agency that doesn't understand, I don't want to waste time, because by the time I've explained, I've probably worked out what that is. That's the main one."* Zainab <br>*"They [my husband's family] really don't quite understand the whole situation, which I think shocked me, because we said he's got to be on a strict diet and his mum came back and said, 'oh yes, I have to do that as well' but she hasn't got kidney disease. And they've said that his brother will donate a kidney. But his brother hasn't once spoken to [my husband] at all about the whole situation. I think they're a little bit naive. We know you can't just donate a kidney, you do have to go through lots of tests and speak to someone if you're mentally ready to do it. Whereas, his brother's just like, yes, I'll give you a kidney. That's all I need to do."* Sarah <br>*"Well I just think, you know, when he's been in renal failure, you know, nobody's really bothered to sort of ask how he is. But now he's got the cancer and the renal failure, it's all focused. And I'm just thinking well actually things aren't any different."* Claire

Relying on yourself	Leveraging existing skills and networks	*"One of my mum friends happens to be an anaesthetist. And she knows the transplant team at [the transplant unit hospital]. […] She was brilliant when I wasn't getting answers from [the primary kidney care hospital], she was just beavering away and finding reassurance.* Olivia<br><br>*"I found the UK Kidney Association myself and I got in contact with them myself. But if only they'd have given us a leaflet at the clinic or something. I just feel that I've got quite a lot of knowledge and I'm not stupid and I can find these things. But there's other people that might be in similar situations that haven't got a clue what to do."* Claire
	"We can't just go out and find it"	*"Because I think sometimes you can feel so upset about things you can't always think where to look."* Rebecca<br><br>*"I can't go see a therapist, I've got kids. You don't really want to do online therapy, for example, because there isn't a time usually when you can. You know, family overhearing you. It's just really the time, also a lot of them cost a lot of money if you want to go and speak to somebody worth speaking to."* Freya<br><br>*"We can't just go out and find it [support]. […] The fact that you can't [go out and get support] because you've got to be here and you've got to plan for getting out and how you do it and when you do it. So that's the other difficulty."* Holly

	When beliefs get in the way	*"I sometimes feel that it is unfair to ask for help or for time off when actually I'm not physically the person that's suffering."* Kate<br><br>*"I suppose I can say that with the consultant that we've got, you know, he always says when we go, he always says, "You know, if there's any problems just ring [my secretary]". And at first I was thinking, oh no, I can't phone her, and I don't want to bother her because she's too busy."* Emily
Support systems can "take the pressure off"	Empathetic support that "instinctively knows" what you need	*"Oh, you know what, it was - and you can't package this - it was the people around me who instinctively knew that I needed something and who just made it easy that I didn't have to ask. Because I didn't even know what I needed for myself at times. And it was the people who were just like, "Why don't you come over for half an hour?" or "Here's some dinner." And those people within them they were all people that were happy to listen, that sort of knew when to keep quiet."* Olivia<br><br>*"Sometimes it's nice when someone asks "how is your brother?", it's something that people that know me, know that is very important for me. […] I really appreciate it because it's part of my life and it's something that's very important for me. It's just the gesture because it tells me, I know what you care about and I want to know."* Sofia

	Support you can count on	*"There is our consultant and there is our nurse. And unless they're on holiday or they're poorly, they are always the constants. And for me that goes a long way because they know us so well that even if things weren't great, I would never be able to tell them any different because they would know."*<br>Kate<br><br>*"**INT:** Now thinking about family, friends, neighbours or colleagues, what's support have you received from them related to your caring role?*<br><br>***RES:** I think emotional support from family and friends, reassurance. Often family members have helped with things like cooking a meal and that just really helps. It takes the pressure off."*<br>Priya<br><br>*"Very good, actually. They [a community organisation] are there, they react. If we ring a phone number we know it's going to get answered. Of course, the monthly meetings, well I don't think we would get by without them to be honest with you."*<br>Holly
